# Bibliography

**Published:** 1903-12-15

**Authors:** 


### BIBLIOGRAPHY.

Another of medical epitome series has just been published by Lea Brothers & Co. This one is on the subject of Anatomy and is compiled by Dr. Henry E. Hale, A.M., M.D., of New York. It is larger than the previous volumes, as it contains nearly four hundred pages. It is surprising how clear the descriptions are made in the brief space given to each subject. The author has also succeeded in introducing the narrative art to such an extent that the little book is really interesting reading. In reading special parts for examination we find ourselves reading on and on into descriptions of other subjects. We predict that this little hand-book will become very popular with students of anatomy. The dental treatment is very brief and contains a statement that may be misleading, but is, however, of no great significance. The statement is made that the molar teeth have from two to *five* fangs. They have two roots in the lower jaw and three in the upper, and when otherwise they are not typical.

—✦—

We have just received a copy of the 1903 Revised Standard Dictionary, and are at a loss to appropriately characterize its general excellence. It is not only the largest and best printed and made single volume we ever saw, but it is one of the greatest achievements of dictionary-making. The publishers started out a few years ago to revise and reprint the old Webster's Dictionary; but because of their habit of doing everything thoroughly they finally produced an entirely new dictionary, which they called "The Standard." It seems to have justified its christening, for it has become a standard reference work with all educators, scholars and literary people. It is remarkable how complete are the definitions on technical subjects. In our own department the definitions are clear and satisfactory. This comes from the employment of well-informed people of all callings to assist in clearly and succinctly defining technical as well as scientific terms. It is certainly a great work, and the Funk & Wagnals Co. has justified its reason for existence, and deserves the good will and patronage of an appreciative public. It defines in a satisfactory manner thousand more words than any dictionary extant. It is besides well and liberally illustrated.

